# The impact of HIV self-testing on risk behaviors among men who have sex with men: a mixed-methods study

**DOI:** 10.3389/fpubh.2024.1369931

**Published:** 2024-02-27

**Authors:** Rong Su, Yi Liu, Peilong Li, Lin Ge, Meizhen Liao, Yong Fu, Xin Song, Duo Shan, Houlin Tang, Dongmin Li

**Affiliations:** ^1^National Center for AIDS/STD Control and Prevention, Chinese Center for Disease Control and Prevention, Beijing, China; ^2^Shandong Provincial Center for Disease Control and Prevention, Ji’nan, China; ^3^Qingdao Municipal Center for Disease Control and Prevention, Qingdao, China

**Keywords:** men who have sex with men, HIV, self-testing, impact, risk behaviors

## Abstract

**Background:**

Men who have sex with men (MSM) have a high prevalence of HIV and a low rate of HIV testing in China. HIV self-testing (HIVST) presents a viable strategy for expanding HIV testing among MSM. However, the impact of HIVST on risk behaviors among MSM remains controversial. Our study sought to ascertain this impact.

**Methods:**

From April 2021 to January 2022, a mixed-methods study was conducted in Qingdao City, employing both quantitative and qualitative methodologies. The quantitative component entailed a cohort study among MSM who had used HIVST. Generalized estimating equations fitting Poisson regressions were used to analyze the changes in risk behaviors of MSM in short time after HIVST (ST-HIVST) and longer time after HIVST (LT-HIVST) compared to before HIVST. Subsequently, we conducted in-depth interviews with 18 MSM who completed the follow-up to delve deeper into the impact of HIVST on MSM.

**Results:**

A total of 410 MSM were recruited in the cohort, of whom 83 were lost to follow-up. Compared to before HIVST, there were no significant changes in risk behaviors in ST-HIVST (*p* > 0.05), while the proportion of recreational drugs abuse (20.7% vs. 33.3%), commercial sex (14.6% vs. 22.9%), and unprotected anal sex (95.9% vs. 98.5%) increased significantly in LT-HIVST (*p* < 0.05). Specific changes varied across demographic characteristics. According to qualitative interviews, MSM might have decreased risk perception and increased risk behaviors after HIVST.

**Conclusion:**

The use of HIVST may promote MSM to engage in risk behaviors. In the future, customized HIVST promotion programs need to be developed to expand HIV testing among MSM and simultaneously control their risk behaviors.

## Introduction

Men who have sex with men (MSM) exhibited a high occurrence of risk behaviors such as multiple sexual partners, unprotected anal sex, and group sex, making them a high-risk population for HIV infection (1–3). UNAIDS estimated that MSM accounted for around 17% of new HIV infections worldwide (4). Globally, the median HIV prevalence among MSM was 7.5%, significantly surpassing that of adult population aged 14–49, which was 0.7% (5). The prevalence of HIV in China remained relatively low in recent years, at 0.1% in 2022, while the proportion of HIV transmission through homosexual sex exhibited an upward trajectory, rising from 9.1% in 2009 to 23.3% in 2020 (6). According to the national HIV sentinel surveillance data from 2022, the HIV prevalence among MSM in China reached 7.0%, with certain regions exceeding 20.0% (7), which indicated a concerning HIV epidemic among MSM in China.

HIV testing is the sole mean of identifying HIV-infected individuals. Expanding HIV testing can facilitate the discovery of HIV-infected individuals, thereby initiating antiviral treatment at an earlier stage and mitigating the consequences of delayed detection (8, 9). Additionally, it is also beneficial to reduce HIV incidence among MSM. A mathematical model for MSM estimated that achieving an 80% HIV testing coverage could reduce the new HIV infections by 70% in 20 years (10). However, the HIV testing rate among MSM was low, due to factors like poor risk perception (11), stigma (12) and limited accessibility to facility-based HIV testing (13). Over the past few years, the HIV testing rate among MSM in China remained at around 60% (14), indicating the need for expanding HIV testing among this population.

The United Nations released 95-95-95 targets for ending AIDS by 2030, with HIV testing as the first target (15). To achieve above targets, countries around the world actively explored strategies to promote HIV testing. In 2016, the WHO recommended using HIV self-testing (HIVST) to expand HIV testing (16). Compared to facility-based HIV testing, HIVST is more convenient (17) and private (18), resulting in higher acceptance rates among MSM. In several areas of China, over half of MSM utilized HIVST (19). Approximately half of MSM who have used HIVST in China were tested for HIV for the first time (20, 21), suggesting the potential of HIVST to reach hidden HIV infections within MSM. And numerous studies confirmed that HIVST could promote facility-based HIV testing (22, 23). Therefore, promoting HIVST is conducive to achieve the 95-95-95 targets in China.

However, there is controversy about the impact of HIVST on risk behaviors among MSM. It was suggested that MSM using HIVST had higher risk awareness, and HIVST could reduce their risk behaviors (24). Others believed that HIVST promoted risk behaviors such as unprotected anal sex (25). The aim of our study was to understand the impact of HIVST on risk behaviors among MSM through a mixed-methods study in Qingdao City, China.

## Materials and methods

### Study site

This study was conducted in Qingdao city. The city was selected based on following reasons: (1) HIV prevalence among MSM in Qingdao was on the rise; (2) Qingdao Center for Disease Control and Prevention (CDC) cooperated well with local non-governmental organizations (NGOs); and (3) local NGOs had close contact with MSM and had vast fieldwork experience.

### Study design

We conducted a mixed-methods study combining quantitative and qualitative designs. The quantitative study was a cohort study with three parts: a baseline survey, a retrospective survey, and a follow-up visit. From April to August 2021, eligible participants were recruited by NGOs using snowball sampling method. We asked them to complete the baseline and retrospective surveys, collecting information about them in the moment and before HIVST, respectively. The follow-up visit was conducted 6 months later, aiming to collect behavioral information of the study participants in a longer time after HIVST. During the study, the local NGOs kept in touch with participants via WeChat and reminded them to engage in the follow-up visit. Subsequently, we conducted in-depth interviews with MSM who completed the follow-up. To ensure the representativeness, the in-depth interviewees were recruited based on the principle of information saturation. We stopped recruiting when no new information relevant to the purpose of this study developed during the interviews.

In order to improve the quality of the data, we selected the staff of local NGOs as investigators. They had close contact with local MSM, who trusted them very much and were more cooperative with their investigation. We trained all the investigators, ensuring that they mastered certain investigation skills. The fieldwork was conducted in a separate room, ensuring the privacy of the participants. Besides, we did not offer monetary incentives to participants, but instead provided free HIV testing and counseling to reduce lost visits.

### Eligibility

MSM enrolled in the baseline survey met the following criteria: (1) be born as male; (2) at least 18 years old; (3) live in Qingdao; (4) had sex with man in the last 6 months; (5) used HIVST in the last 6 months; (6) HIV tested negative at baseline; and (7) be willing to participate and sign an informed consent form.

The in-depth interviewees were recruited from those who had completed the follow-up visit. They were tested negative for HIV. And they agreed that the interviews would be audio-recorded.

### Data collection

Data for the cohort study was collected through face-to-face interviews by NGOs. The following information was collected in each survey: sociodemographic characteristics (age, marital status, registered residence, education, occupation, average monthly income, and sexual orientation), recreational drugs abuse (how to use, where to use, how often to use), sexual behavior (casual sex, commercial sex, unprotected anal sex, group sex, etc.).

The in-depth interviews were conducted by researchers to collect audio-recorded data. The interviews were intended to collect information on the impact of HIVST on MSM, and to explore the reasons. Each interview lasted about 30 min.

### Statistical analysis

At baseline survey, all of the participants had used HIVST during the last 6 months, so we defined this period as short time after HIVST (ST-HIVST). The retrospective survey was taken as before HIVST and the follow-up visit was taken as longer time after HIVST (LT-HIVST).

Cohort study data were entered and verified by two researchers independently using EpiData3.1 software. The statistical analysis was performed using R4.2.3 software. We conducted descriptive analysis using frequency and proportion for the sociodemographic characteristics of the participants. The chi-square test was used to compare the differences in sociodemographic characteristics between the followers and those who lost to follow-up. The outcome variables in this study included various risk behaviors that may facilitate HIV transmission, including recreational drugs abuse, casual sex, commercial sex, unprotected anal sex, and group sex. Generalized estimating equations fitting Poisson regression were used to analyze changes in the outcome in ST-HIVST and LT-HIVST compared to before HIVST. Variables with statistical significance were stratified by sociodemographic characteristics. All statistical tests were two-sided and were statistically significant at *p* < 0.05.

In-depth interview data were transcribed manually from audio recordings into documents. And they were checked by two researchers to ensure completeness and accuracy. After that, we encoded documents with NVivo12.0 software. The codes were discussed and categorized by researchers. The impact of HIVST on MSM was drawn from analyzing and summarizing the codes.

## Results

### Quantitative study

A total of 410 MSM were recruited in the cohort study, of whom 327 completed the follow-up survey and 83 were lost to follow-up. Both two groups mainly consisted of those who were < 40 years old, unmarried, Qingdao household registration, college or higher education, full-time employment, average monthly income ≥5,000 RMB, and homosexual orientation. The difference between the two groups was not statistically significant (*p* > 0.05) (Table 1).

**Table 1:** Comparison of sociodemographic characteristics between follow-up and lost to follow-up groups.

Characteristics	Follow-up *n* (%)	Lost to follow-up *n* (%)	𝜒^2^	*p*
Age group			0.795	0.672
19–29	140 (42.8)	40 (42.8)		
30–39	141 (43.1)	32 (38.6)		
≥40	46 (14.1)	11 (13.3)		
Marital status			0.104	0.748
Unmarried	228 (69.7)	60 (72.3)		
Married/divorced/widowed	99 (30.3)	23 (27.7)		
Registered residence			0.940	0.332
Qingdao	218 (66.7)	50 (60.2)		
Others	109 (33.3)	33 (39.8)		
Education			0.373	0.541
High school or less	84 (25.7)	18 (21.7)		
College or higher	243 (74.3)	65 (78.3)		
Whether having a full-time job			0.003	0.953
Yes	283 (86.5)	71 (85.5)		
No	44 (13.5)	12 (14.5)		
Average monthly income			<0.001	0.993
<5000RMB	151 (46.2)	39 (47.0)		
≥5000RMB	176 (53.8)	44 (53.0)		
Sexual orientation			<0.001	1.000
Homosexual	299 (91.4)	76 (91.6)		
Heterosexual/bisexual	28 (8.6)	7 (8.4)		

#### Changes in risk behaviors

Compared with before HIVST, proportions of recreational drugs abuse (20.7% vs. 21.5%), commercial sex (14.6% vs. 17.3%), and unprotected anal sex (95.9% vs. 96.6%) increased in ST-HIVST. The proportions of casual sex (99.0% vs. 98.0%) and group sex (88.0% vs. 87.3%) decreased. These changes were not statistically significant (*p* > 0.05). The proportion of MSM engaging in each of these risk behaviors increased in LT-HIVST compared to before HIVST. Specifically, the increase in the proportion of recreational drugs abuse (*RR* = 1.198, 95%*CI*: 1.057–1.357), commercial sex (*RR* = 1.263, 95%*CI*: 1.071–1.491), and unprotected anal sex (*RR* = 1.014, 95%*CI*: 1.001–1.028) was statistically significant (*p* < 0.05) (Table 2).

**Table 2:** Change in risk behaviors before, ST- and LT- HIVST.

Variables	Prevalence (%)			*RR* (95%*CI*)	
	Before HIVST	ST-HIVST	LT-HIVST	ST-HIVST vs. Before HIVST	LT-HIVST vs. Before HIVST
Recreational drugs abuse	20.7	21.5	33.3	1.035 (0.794–1.349)	1.198 (1.057–1.357) **
Casual sex	99.0	98.0	98.8	0.990 (0.974–1.007)	1.006 (0.995–1.020)
Commercial sex	14.6	17.3	22.9	1.183 (0.863–1.620)	1.263 (1.071–1.491) **
Unprotected anal sex	95.9	96.6	98.5	1.008 (0.981–1.040)	1.014 (1.001–1.028) *
Group sex	88.0	87.3	89.6	0.992 (0.942–1.040)	0.998 (0.973–1.020)

Risk ratio calculated using generalized estimating model with Poisson regression. *p < 0.05; **p < 0.01. RR, risk ratio; CI, confidence interval; HIVST, HIV self-testing.

#### Changes in recreational drugs abuse

Compared with before HIVST, MSM aged 19–29 years (21.8% vs. 20.6%), married/divorced/widowed (19.0% vs. 18.0%), registered residence in Qingdao (20.8% vs. 20.1%), having average monthly income ≥5,000 RMB (22.0% vs. 21.5%), and reporting homosexual orientation (18.9% vs. 17.1%) experienced a decrease in proportion in ST-HIVST, while the remaining subgroups showed an increase. None of these changes were statistically significant (*p* > 0.05). In LT-HIVST, proportions in all subgroups increased compared to before HIVST. Notably, the proportion among MSM aged 30–39 years (*RR* = 1.278, 95%*CI*: 1.056–1.547), unmarried (*RR* = 1.226, 95%*CI*: 1.062–1.416), registered residence in Qingdao (*RR* = 1.174, 95%*CI*: 1.008–1.370) or other locations (*RR* = 1.247, 95%*CI*: 1.003–1.550), with a college or higher education (*RR* = 1.187, 95%*CI*: 1.036–1.360), having a full-time job (*RR* = 1.185, 95%*CI*: 1.042–1.350), with an average monthly income <5,000 RMB (*RR* = 1.223, 95%*CI*: 1.012–1.477), and reporting homosexual (*RR* = 1.181, 95%*CI*: 1.038–1.342) increased significantly (*p* < 0.05) (Table 3).

**Table 3:** Changes in recreational drugs abuse before, ST- and LT- HIVST.

Group	Prevalence (%)			*RR* (95%*CI*)	
	Before HIVST	ST- HIVST	LT- HIVST	ST-HIVST vs. Before HIVST	LT-HIVST vs. Before HIVST
Age group					
19–29	21.8	20.6	31.9	0.945 (0.638–1.400)	1.144 (0.949–1.379)
30–39	20.2	23.1	37.1	1.142 (0.759–1.720)	1.278 (1.056–1.547) *
≥40	18.5	19.3	26.9	1.042 (0.482–2.250)	1.134 (0.786–1.640)
Marital status					
Unmarried	21.5	22.9	36.1	1.068 (0.787–1.450)	1.226 (1.062–1.416) **
Married/divorced/widowed	19.0	18.0	27.0	0.949 (0.560–1.610)	1.123 (0.875–1.440)
Registered residence					
Qingdao	20.8	20.1	32.6	0.968 (0.694–1.350)	1.174 (1.008–1.370) *
Others	20.6	23.9	34.9	1.164 (0.752–1.800)	1.247 (1.003–1.550) *
Education					
High school or less	14.4	15.7	25.3	1.088 (0.568–2.080)	1.239 (0.916–1.677)
College or higher	22.9	23.4	36.1	1.022 (0.766–1.360)	1.187 (1.036–1.360) *
Whether having a full-time job					
Yes	22.4	23.2	35.0	1.032 (0.787–1.350)	1.185 (1.042–1.350) **
No	10.3	10.7	22.7	1.036 (0.355–3.020)	1.320 (0.832–2.100)
Average monthly income					
<5000RMB	19.3	21.5	32.5	1.114 (0.749–1.660)	1.223 (1.012–1.477) *
≥5000RMB	22.0	21.5	34.1	0.975 (0.683–1.390)	1.178 (0.998–1.391)
Sexual orientation					
Homosexual	20.9	21.9	33.6	1.046 (0.794–1.380)	1.181 (1.038–1.342) *
Heterosexual/bisexual	18.9	17.1	31.0	0.906 (0.337–2.430)	1.474 (0.869–2.500)

Risk ratio calculated using generalized estimating model with Poisson regression. *p < 0.05; **p < 0.01. RR, risk ratio; CI, confidence interval; HIVST, HIV self-testing.

#### Changes in commercial sex

The proportion of commercial sex increased in all subgroups in ST-HIVST compared to before HIVST. None of these changes were statistically significant (*p* > 0.05). In LT-HIVST, the proportion increased in most subgroups, except for MSM reporting a homosexual orientation. Statistically significant increases were observed among those aged 30–39 years (*RR* = 1.499, 95%*CI*: 1.110–2.027), unmarried (*RR* = 1.339, 95%*CI*: 1.078–1.664), registered residence in Qingdao (*RR* = 1.289, 95%*CI*: 1.052–1.580), with college or higher education (*RR* = 1.314, 95%*CI*: 1.074–1.609), having a full-time job (*RR* = 1.270, 95%*CI*: 1.062–1.519), with an average monthly income ≥5,000 RMB (*RR* = 1.392, 95%*CI*: 1.114–1.741), and reporting a homosexual orientation (*RR* = 1.293, 95%*CI*: 1.087–1.538) (*p* < 0.05) (Table 4).

**Table 4:** Changes in commercial sex before, ST- and LT- HIVST.

Group	Prevalence (%)			*RR* (95%*CI*)	
	Before HIVST	ST- HIVST	LT- HIVST	ST-HIVST vs. Before HIVST	LT-HIVST vs. Before HIVST
Age group					
19–29	14.5	17.2	20.0	1.187 (0.743–1.900)	1.176 (0.910–1.520)
30–39	11.0	13.9	22.1	1.256 (0.709–2.230)	1.499 (1.110–2.027) **
≥40	25.9	28.1	32.7	1.083 (0.586–2.000)	1.082 (0.794–1.470)
Marital status					
Unmarried	12.8	15.3	21.1	1.193 (0.795–1.790)	1.339 (1.078–1.664) **
Married/divorced/widowed	19.0	22.1	27.0	1.164 (0.709–1.910)	1.150 (0.893–1.480)
Registered residence					
Qingdao	14.1	15.4	23.4	1.162 (0.779–1.730)	1.289 (1.052–1.580) *
Others	15.6	19.0	22.0	1.219 (0.730–2.030)	1.214 (0.911–1.617)
Education					
High school or less	19.2	21.6	25.3	1.122 (0.653–1.930)	1.159 (0.869–1.550)
College or higher	13.1	15.9	22.1	1.217 (0.827–1.790)	1.314 (1.074–1.609) **
Whether having a full-time job					
Yes	14.8	17.2	23.0	1.166 (0.831–1.640)	1.270 (1.062–1.519) **
No	13.8	17.9	22.7	1.295 (0.551–3.040)	1.222 (0.790–1.890)
Average monthly income					
<5000RMB	15.1	18.3	18.8	1.213 (0.774–1.900)	1.116 (0.869–1.430)
≥5000RMB	14.2	16.4	26.6	1.156 (0.743–1.800)	1.392 (1.114–1.741) **
Sexual orientation					
Homosexual	14.2	17.1	23.5	1.201 (0.860–1.680)	1.293 (1.087–1.538) **
Heterosexual/bisexual	18.9	20.0	17.2	1.057 (0.413–2.710)	0.983 (0.560–1.730)

Risk ratio calculated using generalized estimating model with Poisson regression. *p < 0.05; **p < 0.01. RR, risk ratio; CI, confidence interval; HIVST, HIV self-testing.

#### Changes in unprotected anal sex

Compared to before HIVST, the proportion of unprotected anal sex increased in all subgroups in ST- and LT- HIVST, except for individuals reporting a homosexual orientation, who experienced a decrease in ST-HIVST. And the changes observed in ST-HIVST were not statistically significant in any of the subgroups (*p* > 0.05). However, in LT-HIVST, the proportion among those aged 19–29 years (*RR* = 1.020, 95%*CI*: 1.000–1.040) and having a full-time job (*RR* = 1.015, 95%*CI*: 1.001–1.029) statistically increased (*p* < 0.05) (Table 5).

**Table 5:** Changes in unprotected anal sex before, ST- and LT- HIVST.

Group	Prevalence (%)			*RR* (95%*CI*)	
	Before HIVST	ST-HIVST	LT-HIVST	ST-HIVST vs. Before HIVST	LT-HIVST vs. Before HIVST
Age group					
19–29	96.4	97.2	99.3	1.009 (0.972–1.050)	1.020 (1.000–1.040) *
30–39	95.1	96.0	97.9	1.009 (0.963–1.060)	1.009 (0.988–1.030)
≥40	96.3	96.5	98.1	1.000 (0.932–1.080)	1.014 (0.976–1.050)
Marital status					
Unmarried	96.2	96.9	98.7	1.007 (0.976–1.040)	1.016 (1.000–1.032)
Married/divorced/widowed	95.0	95.9	98.0	1.009 (0.955–1.070)	1.011 (0.986–1.040)
Registered residence					
Qingdao	97.8	97.8	99.5	1.000 (0.975–1.030)	1.012 (1.000–1.024)
Others	92.2	94.4	96.3	1.024 (0.961–1.090)	1.020 (0.988–1.050)
Education					
High school or less	97.1	99.0	100.0	1.020 (0.981–1.060)	1.012 (0.995–1.030)
College or higher	95.4	95.8	98.0	1.004 (0.970–1.040)	1.015 (0.998–1.033)
Whether having a full-time job					
Yes	96.0	96.6	98.6	1.006 (0.977–1.040)	1.015 (1.001–1.029) *
No	94.8	96.4	97.7	1.017 (0.940–1.100)	1.022 (0.973–1.050)
Average monthly income					
<5000RMB	99.0	99.5	100.0	1.005 (0.988–1.020)	1.003 (0.997–1.010)
≥5000RMB	93.1	94.1	97.1	1.010 (0.962–1.060)	1.024 (0.999–1.050)
Sexual orientation					
Homosexual	95.7	96.5	98.3	1.009 (0.980–1.040)	1.014 (1.000–1.028)
Heterosexual/bisexual	97.3	97.1	100.0	0.998 (0.923–1.080)	1.018 (0.983–1.060)

Risk ratio calculated using generalized estimating model with Poisson regression. *p < 0.05; **p < 0.01. RR, risk ratio; CI, confidence interval; HIVST, HIV self-testing.

### In-depth interview

We interviewed 18 MSM, including 2 money boy (MB), 3 students, 11 staff, and 2 freelancers. The age of the interviewees ranged from 20 to 47 years, with a median age of 28.5 (26.0–34.0) years. They were predominantly unmarried, had a college education or higher, earned a monthly income of ≥5,000 RMB, and reporting a homosexual orientation (Table 6).

**Table 6:** Characteristics of in-depth interviewees.

Characteristics	Frequency	Percentage (%)
Median age (IQR)	28.5 (26.0–34.0)	
Occupation		
MB	2	11.1
Student	3	16.7
Staff	11	61.1
Freelancer	2	11.1
Marital status		
Unmarried	15	83.3
Married/divorced/widowed	3	16.7
Education		
High school or less	8	44.4
College or higher	10	55.6
Average monthly income		
<5000RMB	7	38.9
≥5000RMB	11	61.1
Sexual orientation		
Homosexual	13	72.2
Heterosexual/bisexual	5	27.8

IQR, interquartile range; MB, money boy.

#### Changes in risk perception

After HIVST, the HIV risk perception of MSM might decreased. Half of the interviewees reported that a negative result of HIVST equaled to no risk of HIV infection.

“Well, as I rely on doing this (MB) to earn money, I’m definitely afraid of getting infected. I’ll provide them (guests) with that reagent (HIVST), and have a test with them before sex. If the result appears only one bar, OK, come on…In fact, MSM are very cautious now, and some of them even bring their own reagents for me to have a test” (ID4, 28 years old, unmarried, MB).

“I mostly have sex with my boyfriend and occasionally go out on dates, so my risk of HIV infection was very low. Now I have tested negative, it must be risk-free” (ID12, 27 years old, unmarried, staff).

“Hmm, I am confident that I have no risk, cause that’s negative. Besides, at my age, having a wife and children, the risk (of HIV infection) is virtually non-existent. Because I cannot play like a young man” (ID16, 43 years old, married, freelancer).

Some of the interviewees indicated that HIVST was just a test and that it would not affect their risk of HIV infection.

“You know. The risk of infection depends on behavior. This is just a test, cannot affect anything” (ID13, 26 years old, unmarried, staff).

“It’s just a way to jab you and see how many stripes you have. After that, if you are at risk, you are still going to get infected” (ID18, 47 years old, divorced, MB).

#### Changes in risk behaviors

Some MSM stated that they used HIVST with the specific intention of engaging in risk behaviors. This group often interpreted a negative test result as an opportunity to engage in risk behaviors, aiming for enhanced sexual experiences or increased income. Consequently, the risk behavior of HIV infection among MSM increased after HIVST.

“I often seek out 18-year-old MB. When I intend to have sex with them, I will have a test with them together. I usually carry a substantial number of HIVST kits with me…To be honest, I do the test just for the date. If the results are fine, then I can have a date with no worries” (ID2, 28 years old, unmarried, staff).

“The charge for not using condoms and multiple sex is definitely higher. So, after the test, these behaviors are definitely increased. After all, I am involved in this profession (MB), the desire to maximize earnings is a natural inclination” (ID4, 28 years old, unmarried, MB).

“(The use of condoms) Absolutely less. In all honesty, nobody enjoys using condoms, cause they are a pain in the ass, they are uncomfortable, and they cost money” (ID9, 34 years old, unmarried, staff).

Several MSM stated that HIVST had no effect on their behaviors due to having regular sexual partners or families.

“I really care about my health, and I exclusively engage in sexual activities with my boyfriend, regardless of whether or not I’ve used HIVST before…We do not use condoms, mainly because we have been in a committed relationship for several years, both tested negative for HIV, and trust each other” (ID10, 36 years old, unmarried, freelancer).

“I have sex two or three times a month, and my sexual desire is not particularly high. Consequently, getting tested or not does not significantly influence me. And I always ask my sexual partner to wear a condom. If they refuse, I will abstain from the relationship” (ID14, 20 years old, unmarried, student).

“Cause I’m worried about going to the hospital and being caught by my family, I use HIVST kits regularly. It has no effect on my behaviors. On occasion, when the need arises, I engage in dating and consistently use condoms. Because I never use condoms with my wife, I must prevent getting infected outside in case of transmitting it to my family” (ID17, 36 years old, married, staff).

## Discussion

In the cohort study, 410 MSM were recruited. Of these, 83 were lost to follow-up due to the lack of monetary incentives and the long follow-up period. There was no statistically significant difference in the sociodemographic characteristics of the lost individuals and the followers. Thus, absence from the follow-up survey had no effect on the results of the quantitative study.

Evidence shows an association between HIVST use and risk awareness (19). Our findings indicated that, in comparison to before HIVST, the proportion of MSM engaging in casual and group sex decreased in ST-HIVST, although the result did not reach statistical significance. This suggested that MSM might exhibit increased risk awareness in ST-HIVST, potentially resulting in a lower proportion of risk behaviors than before HIVST. In contrast to the findings of Tang (26), our study found a decrease in risk perception and an increase in risk behaviors among MSM in LT-HIVST. This discrepancy may be attributed to differences in study design. Tang’s study included a follow-up of MSM at 3 months after enrollment, allowing for the capture of short-term behavioral changes. In contrast, our study’s follow-up was conducted after 6 months, focusing on longer-term changes.

The World Drug Report 2022 highlighted a concerning global trend of increasing recreational drugs use and the subsequent adverse health effects (27). Specifically, due to its ability to relax the anal sphincter and alleviate pain during anal intercourse, recreational drugs are extensively used by MSM (28). The utilization of recreational drugs was associated with a heightened risk of HIV infection among MSM (29, 30). In our study, the proportion of MSM abusing recreational drugs was 20.7% before HIVST, and significantly increased to 33.3% in LT-HIVST. These findings suggested that HIVST might promote recreational drugs abuse among MSM, consequently increasing their risk of HIV infection. Compared to the control of heroin and other conventional drugs, the oversight of recreational drugs in China is comparatively lax, enabling MSM to readily access it through various means. To curtail recreational drugs abuse among MSM and consequently reduce the risk of HIV infection, it is imperative for authorities to implement stricter measures to limit the circulation of recreational drugs.

Acquiring HIVST often necessitates the ability to pay, resulting in a higher percentage of MSM who have ever used HIVST being in full-time employment and with higher average monthly incomes. Additionally, among the various risk behaviors of MSM, commercial sex also associated with economic status (31). Our research revealed that the proportion of MSM involved in commercial sex increased in LT-HIVST compared to before HIVST. Combined with in-depth interviews, it was found that this increase could be attributed to the enhanced accessibility of HIV testing facilitated by the promotion of HIVST. Several MSM were inclined to utilize HIVST for the purpose of engaging in commercial sex while ensuring safety, aligning with the findings of Mujugira (32). These results indicated, on one hand, that MSM still face a notable risk of HIV infection. On the other hand, they also reflected the success of our strategy to expand HIV testing, and awareness of HIV testing among MSM improved to a certain extent.

As we all know, HIV primarily spreads through sexual contact, and consistent condom usage is an effective preventive measure against HIV infection (33, 34). In 2016, China CDC explicitly advocated for the promotion of condom among MSM to mitigate their risk behaviors, such as unprotected anal intercourse (35). However, MSM’s pursuit of sexual sensation, combined with limited self-protective awareness, resulted in a low proportion of consistent condom use during anal sex (36). Our study found that the proportion of MSM engaging in unprotected anal intercourse was about 95% before HIVST, and this proportion increased significantly in LT-HIVST. In-depth interviews revealed that this change could still be attributed to MSM prioritizing the sexual experience, with HIVST potentially lowering their risk awareness and consequently promoting unprotected anal sex. It is indicated the importance of intensifying efforts to promote condom usage.

A large number of MSM in China are married to women due to traditional beliefs about marriage and privacy concerns (37). This group constituted approximately 1/3 of the population in our study. They exhibited a low proportion of condom use during sex with their wife, serving as a bridge for HIV transmission from the homosexual to heterosexual population (38, 39). From in-depth interviews, we found that married MSM often perceived HIVST as a routine form of HIV testing. Therefore, there were no significant changes in risk behaviors among this group after HIVST. In contrast, unmarried MSM frequently identified a negative result as a green light for engaging in risk behaviors. Consequently, we observed an increase in risk behaviors among this subgroup in LT-HIVST.

The HIVST was more prevalent among individuals with higher levels of education (40). In our study, 74.3% of the subjects involved in follow-up had a college education or higher. Compared with before HIVST, this educated population exhibited an increased proportion of recreational drugs abuse and commercial sex in LT-HIVST. The reason might be the higher risk perception among those with higher education (41), who often utilized HIVST before engaging in risk behaviors, thus leading to an increase in risk behaviors in LT-HIVST. It is worth noting that HIVST has a window period, and a negative result does not entirely rule out HIV infection (12). It showed that MSM had insufficient knowledge about HIVST. In the future, there is a pressing need to enhance education and training on HIVST-related knowledge within this population to minimize the risk of HIV infection.

Several limitations also exist in our study. Firstly, we recruited study participants through NGOs, and MSM better connected to NGOs were more likely to be included. This subgroup of MSM may possess greater knowledge about HIVST, exhibit heightened risk awareness, and experience fewer increases in risk behaviors after HIVST. Consequently, the overall increase in risk behaviors may be underestimated to some extent. Nonetheless, given the hidden nature of MSM, relying on NGOs was essential to recruit these concealed groups and ensure the results’ representativeness. Secondly, we did not set up a strict control group, but instead compared behaviors before, ST- and LT- HIVST. The changes of MSM in our study may be accompanied by changes among MSM who did not use HIVST. Thus, the changes in risk behaviors among MSM in our study cannot be exclusively attributed to the use of HIVST. More rigorous cohort studies are required to clarify the impact of HIVST on MSM. Finally, this study used a non-randomized sampling method and the results should to be generalized cautiously.

## Conclusion

Within the context of expanding HIV testing, there is a growing trend in the utilization of HIVST, yet the potential impact of HIVST on the behaviors of MSM is often overlooked. This study showed that the use of HIVST could diminish risk awareness and encourage risk behaviors among MSM, including recreational drugs abuse, commercial sex, and unprotected anal sex. In response to these findings, it is imperative to formulate tailored HIVST promotion initiatives designed to enhance HIV testing rates within MSM, while concurrently mitigating the risk behaviors. In addition, more rigorous cohort studies are needed to provide comprehensive clarification regarding the impact of HIVST on risk behaviors among MSM.

## Data availability statement

The data analyzed in this study is subject to the following licenses/restrictions: the datasets presented in this article are not readily available because of privacy and ethical concerns. Requests to access these datasets should be directed to the corresponding author.

## Ethics statement

The studies involving humans were approved by the Ethics Committee of National Center for AIDS/STD Control and Prevention, Chinese Center for Disease Control and Prevention. The studies were conducted in accordance with the local legislation and institutional requirements. The participants provided their written informed consent to participate in this study.

## Author contributions

RS: Conceptualization, Data curation, Formal analysis, Investigation, Methodology, Project administration, Supervision, Writing – original draft, Writing – review & editing. YL: Data curation, Formal analysis, Investigation, Project administration, Supervision, Writing – review & editing. PL: Data curation, Investigation, Project administration, Supervision, Writing – review & editing. LG: Investigation, Methodology, Project administration, Supervision, Writing – review & editing. ML: Data curation, Investigation, Project administration, Supervision, Writing – review & editing. YF: Data curation, Investigation, Project administration, Supervision, Writing – review & editing. XS: Investigation, Methodology, Project administration, Supervision, Writing – review & editing. DS: Data curation, Formal analysis, Investigation, Project administration, Supervision, Writing – review & editing. HT: Investigation, Project administration, Resources, Supervision, Writing – review & editing. DL: Conceptualization, Funding acquisition, Investigation, Methodology, Project administration, Resources, Supervision, Writing – review & editing.

## References

[1] Shan D Ning Z Yu MH Zheng H Yang J Gong H. HIV incidence and risk factors among transgender women and cisgender men who have sex with men in two cities of China: a prospective cohort study. Infect Dis Poverty. (2022) 11:26. doi: 10.1186/s40249-022-00947-3, PMID: 35256001 PMC8900389

[2] Bowring AL Veronese V Doyle JS Stoove M Hellard M. HIV and sexual risk among men who have sex with men and women in Asia: a systematic review and meta-analysis. AIDS Behav. (2016) 20:2243–65. doi: 10.1007/s10461-015-1281-x, PMID: 26781871

[3] Chen J Fan H Chen HL Yao FF. Correlates of group sex participation among men who have sex with men in Chongqing, Southwestern China. BMC Public Health. (2021) 21:561. doi: 10.1186/s12889-021-10607-0, PMID: 33752635 PMC7983368

[4] UNAIDS. Worldwide, more than half of new HIV infections now among key populations and their sexual partners. (2019). Available at: https://www.unaids.org/en/resources/presscentre/featurestories/2019/november/20191105_key-populations (Accessed Nov 5, 2019).

[5] UNAIDS. Global HIV & AIDS statistics — Fact sheet 2023. (2023). Available at: https://www.unaids.org/en/resources/fact-sheet (Accessed Jul 31, 2023).

[6] He N. Research progress in the epidemiology of HIV/AIDS in China. China CDC Wkly. (2021) 3:1022–30. doi: 10.46234/ccdcw2021.249, PMID: 34888119 PMC8633551

[7] Han MJ. Analysis of the epidemic of HIV/AIDS in China and prospects for prevention and control. Chin J AIDS STD. (2023) 29:247–50. doi: 10.13419/j.cnki.aids.2023.03.01

[8] Croxford S Kitching A Desai S Kall M Edelstein M Skingsley A. Mortality and causes of death in people diagnosed with HIV in the era of highly active antiretroviral therapy compared with the general population: an analysis of a national observational cohort. Lancet Public Health. (2017) 2:e35–46. doi: 10.1016/S2468-2667(16)30020-2, PMID: 29249478

[9] WHO. New HIV/AIDS political declaration seeks to end inequalities and get on track to end AIDS by 2030. (2021). Available at: https://www.who.int/news/item/11-06-2021-new-hiv-aids-political-declaration-seeks-to-end-inequalities-and-get-on-track-to-end-aids-by-2030 (Accessed Jun 11, 2021).

[10] Caro-Vega Y Del Rio C Lima VD Lopez-Cervantes M Crabtree-Ramirez B Bautista-Arredondo S. Estimating the impact of earlier ART initiation and increased testing coverage on HIV transmission among men who have sex with men in Mexico using a mathematical model. PLoS One. (2015) 10:e0136534. doi: 10.1371/journal.pone.0136534, PMID: 26302044 PMC4547810

[11] Sun SF Whiteley L Brown LK. HIV testing among Chinese men who have sex with men: the roles of HIV knowledge, online social life, and sexual identity concerns. AIDS Behav. (2020) 24:437–49. doi: 10.1007/s10461-019-02471-2, PMID: 30924064 PMC6765462

[12] Liu Y Wu GH Lu RR Ou R Hu L Yin YP. Facilitators and barriers associated with uptake of HIV self-testing among men who have sex with men in Chongqing, China: a cross-sectional survey. Int J Environ Res Public Health. (2020) 17:1634. doi: 10.3390/ijerph17051634, PMID: 32138263 PMC7084434

[13] He L Pan XH Yang JZ Ma QA Jiang J Wang W. HIV risk behavior and HIV testing among rural and urban men who have sex with men in Zhejiang Province, China: a respondent-driven sampling study. PLoS One. (2020) 15:e0231026. doi: 10.1371/journal.pone.0231026, PMID: 32240244 PMC7117739

[14] UNAIDS. The key populations atlas (2023). Available at:(https://kpatlas.unaids.org/dashboard).

[15] UNAIDS. Political declaration on HIV and AIDS: Ending inequalities and getting on track to end AIDS by 2030. (2021). Available at:(https://www.unaids.org/en/resources/documents/2021/2021_political-declaration-on-hiv-and-aids).

[16] WHO. Guidelines on HIV self-testing and partner notification: supplement to consolidated guidelines on HIV testing services. Geneva: WHO (2016). 27977094

[17] Li JJ Marley G Zhang Y Chen YT Tang WM Rongbin Y. Determinants of recent HIV self-testing uptake among men who have sex with men in Jiangsu Province, China: an online cross-sectional survey. Front Public Health. (2021) 9:736440. doi: 10.3389/fpubh.2021.736440, PMID: 34790641 PMC8591127

[18] Fan S Liu ZQ Luo ZZ Yu MH Ouyang L Gong H. Effect of availability of HIV self-testing on HIV testing frequency among men who have sex with men attending university in China (UniTest): protocol of a stepped-wedge randomized controlled trial. BMC Infect Dis. (2020) 20:149. doi: 10.1186/s12879-020-4807-4, PMID: 32070297 PMC7029612

[19] Marley G Fu GF Zhang Y Li JJ Tucker JD Tang WM. Willingness of Chinese men who have sex with men to use smartphone-based electronic readers for HIV self-testing: web-based cross-sectional study. J Med Internet Res. (2021) 23:e26480. doi: 10.2196/26480, PMID: 34806988 PMC8663451

[20] Qin YL Tang WM Nowacki A Mollan K Reifeis SA Hudgens MG. Benefits and potential harms of human immunodeficiency virus self-testing among men who have sex with men in China: an implementation perspective. Sex Transm Dis. (2017) 44:233–8. doi: 10.1097/olq.0000000000000581, PMID: 28282650 PMC5347468

[21] Xiu XF Qin YY Bao YG Chen YK Wu H Huang XJ. The practice and potential role of HIV self-testing in China: systematic review and Meta-analysis. JMIR Public Health Surveill. (2022) 8:e41125. doi: 10.2196/41125, PMID: 36459393 PMC9758640

[22] Zhang C Li XH Brecht ML Koniak-Griffin D. Can self-testing increase HIV testing among men who have sex with men: a systematic review and meta-analysis. PLoS One. (2017) 12:e0188890. doi: 10.1371/journal.pone.0188890, PMID: 29190791 PMC5708824

[23] Zhang C Li XH Koniak-Griffin D Goldsamt LA Zhou J. Effectiveness of self-testing kits availability on improving HIV testing frequency for chinese men who have sex with men and their sexual partners: a protocol for a multicenter randomised controlled trial. BMJ Open. (2018) 8:e024423. doi: 10.1136/bmjopen-2018-024423, PMID: 30552280 PMC6303590

[24] Frasca T Balan I Ibitoye M Valladares J Dolezal C Carballo-Diéguez A. Attitude and behavior changes among gay and bisexual men after use of rapid home HIV tests to screen sexual partners. AIDS Behav. (2014) 18:950–7. doi: 10.1007/s10461-013-0630-x, PMID: 24077975 PMC3969408

[25] Wood BR Ballenger C Stekler JD. Arguments for and against HIV self-testing. HIV AIDS (Auckl). (2014) 6:117–26. doi: 10.2147/hiv.S49083, PMID: 25114592 PMC4126574

[26] Tang WM Huang WT Lu HD Cao BL Wu D Ong J. What happens after HIV self-testing? Results from a longitudinal cohort of Chinese men who have sex with men. BMC Infect Dis. (2019) 19:807. doi: 10.1186/s12879-019-4455-8, PMID: 31521123 PMC6744670

[27] UNODC. World drug report 2022. (2022). Available at: https://www.unodc.org/res/wdr2022/MS/WDR22_Booklet_4.pdf (Accessed Jun 27, 2022).

[28] Hall TM Shoptaw S Reback CJ. Sometimes poppers are not poppers: huffing as an emergent health concern among MSM substance users. J Gay Lesbian Ment Health. (2014) 19:118–21. doi: 10.1080/19359705.2014.973180, PMID: 25893032 PMC4399803

[29] Shan D Yu MH Yang J Zhuang MH Ning Z Liu H. Correlates of HIV infection among transgender women in two Chinese cities. Infect Dis Poverty. (2018) 7:123. doi: 10.1186/s40249-018-0508-2, PMID: 30509315 PMC6276265

[30] Jiang HB Li J Tan ZM Chen XB Cheng WB Gong X. Syndemic factors and HIV risk among men who have sex with men in Guangzhou, China: evidence from synergy and moderated analyses. Arch Sex Behav. (2020) 49:311–20. doi: 10.1007/s10508-019-01488-x, PMID: 31617111

[31] Cai YM Song YJ Liu H Hong FC. Factors associated with commercial sexual behavior among men who have sex with men in Shenzhen, China, in 2011–2015. Chin J Prev Med. (2016) 50:943–8. doi: 10.3760/cma.j.issn.0253-9624.2016.11.005, PMID: 27903355

[32] Mujugira A Nakyanzi A Kasiita V Kamusiime B Nalukwago GK Nalumansi A. HIV self-testing and oral pre-exposure prophylaxis are empowering for sex workers and their intimate partners: a qualitative study in Uganda. J Int AIDS Soc. (2021) 24:e25782. doi: 10.1002/jia2.25782, PMID: 34473405 PMC8412089

[33] Holmes KK Levine R Weaver M. Effectiveness of condoms in preventing sexually transmitted infections. Bull World Health Organ. (2004) 82:454–61. PMID: 15356939 PMC2622864

[34] Cayley WE Jr. Effectiveness of condoms in reducing heterosexual transmission of HIV. Am Fam Physician. (2004) 70:1268–9. PMID: 15508535

[35] National Center for AIDS/STD Control and Prevention, Chinese Center for Disease Control and Prevention. Guidelines for HIV prevention interventions among men who have sex with men. (2016). Available at: https://ncaids.chinacdc.cn/fzyw_10256/jsgf/201804/t20180419_164176.htm (Accessed Sep 13, 2016).

[36] Li YZ Xu JJ Qian HZ You BX Zhang J Zhang JM. High prevalence of HIV infection and unprotected anal intercourse among older men who have sex with men in China: a systematic review and meta-analysis. BMC Infect Dis. (2014) 14:531. doi: 10.1186/1471-2334-14-531, PMID: 25287717 PMC4287343

[37] Wu WZ Yan XC Zhang XX Goldsamt L Chi YY Huang DP. Potential HIV transmission risk among spouses: marriage intention and expected extramarital male-to-male sex among single men who have sex with men in Hunan, China. Sex Transm Infect. (2020) 96:151–6. doi: 10.1136/sextrans-2018-053906, PMID: 31171593 PMC7035676

[38] Mor Z Davidovich U Bessudu-Manor N Mcfarlane M Feldshtein G Chemtob D. High-risk behaviour in steady and in casual relationships among men who have sex with men in Israel. Sex Transm Infect. (2011) 87:532–7. doi: 10.1136/sextrans-2011-050048, PMID: 21917699

[39] Li HC Lau JT Holroyd E Yi H. Sociocultural facilitators and barriers to condom use during anal sex among men who have sex with men in Guangzhou, China: an ethnographic study. AIDS Care. (2010) 22:1481–6. doi: 10.1080/09540121.2010.482121, PMID: 20824556

[40] Hong H Shi HB Jiang HB Dong HJ Shen YL. Prevalence and associated factors of HIV self-testing among men who have sex with men in Ningbo, China: a cross-sectional study. AIDS Res Ther. (2021) 18:14. doi: 10.1186/s12981-021-00339-x, PMID: 33879191 PMC8056656

[41] Wong HT Tam HY Chan DP Lee SS. Usage and acceptability of HIV self-testing in men who have sex with men in Hong Kong. AIDS Behav. (2015) 19:505–15. doi: 10.1007/s10461-014-0881-1 25145608

